# The Effect of Socioeconomic Factors on Spatiotemporal Patterns of PM_2.5_ Concentration in Beijing–Tianjin–Hebei Region and Surrounding Areas

**DOI:** 10.3390/ijerph17093014

**Published:** 2020-04-26

**Authors:** Wenting Wang, Lijun Zhang, Jun Zhao, Mengge Qi, Fengrui Chen

**Affiliations:** 1 Key Laboratory of Geospatial Technology for the Middle and Lower Yellow River Regions, Ministry of Education/Collaborative Innovation Center of Yellow River Civilization, Henan University, Kaifeng 475004, China; 2 College of Environmental and Planning, Henan University, Kaifeng 475004, China; 3 South-to-North Water Diversion Middle Route Information Technology Co., Ltd., Beijing 100038, China

**Keywords:** PM_2.5_, socioeconomic factors, spatiotemporal patterns, spatiotemporal heterogeneous, spatial panel Dubin model

## Abstract

The study investigated the spatiotemporal evolution of PM_2.5_ concentration in the Beijing–Tianjin–Hebei region and surrounding areas during 2015–2017, and then analyzed its socioeconomic determinants. First, an estimation model considering spatiotemporal heterogeneous relationships was developed to accurately estimate the spatial distribution of PM_2.5_ concentration. Additionally, socioeconomic determinants of PM_2.5_ concentration were analyzed using a spatial panel Dubin model, which aimed to improve the robustness of the model estimation. The results demonstrated that: (1) The proposed model significantly increased the estimation accuracy of PM_2.5_ concentration. The mean absolute error and root-mean-square error were 9.21 μg/m^3^ and 13.10 μg/m^3^, respectively. (2) PM_2.5_ concentration in the study area exhibited significant spatiotemporal changes. Although the PM_2.5_ concentration has declined year by year, it still exceeded national environmental air quality standards. (3) The per capita GDP, urbanization rate and number of industrial enterprises above the designated size were the key factors affecting the spatiotemporal distribution of PM_2.5_ concentration. This study provided scientific references for comprehensive PM_2.5_ pollution control in the study area.

## 1. Introduction

Atmospheric pollution significantly influences human health, climatic environment, and sustainable urban development [1,2,3,4]. According to a World Health Organization (WHO) report in 2014, atmospheric pollution causes more than seven million deaths worldwide each year [5]. A recent study based on the Global Exposure Mortality Model estimated that 8.9 million people died globally in 2015 [6]. With China’s rapid economic, industrial, and urban development, atmospheric pollution has become an increasing problem. East China, especially the Beijing–Tianjin–Hebei region, has witnessed frequent occurrences of severe haze since 2013. The Beijing–Tianjin–Hebei region is responsible for the majority of North China’s economic development, and coordinated development in this region is one of three national strategies in China [7]. Therefore, comprehensive atmospheric pollution governance in the region has attracted wide attention from China’s government. PM_2.5_ (atmospheric particulate matter with a diameter of less than 2.5 µm) is the primary cause of haze. Twenty-eight cities in the Beijing–Tianjin–Hebei region and surrounding areas are considered to be the main transmission channels of atmospheric pollution in the region. Hence, accurate interpretation of spatiotemporal distribution and evolution of PM_2.5_ concentrations in the Beijing–Tianjin–Hebei region and surrounding areas, as well as a recognition of the primary influencing factors of PM_2.5_ concentrations, have important theoretical and practical significance to atmospheric pollution control.

An accurate estimation of the spatial distribution of PM_2.5_ concentration is a prerequisite for determining its determinants. Traditionally, the spatial distribution of PM_2.5_ concentration is generated through spatially interpolating ground PM_2.5_ observations [8,9]. Although the ground observations are accurate, the observation stations are limited in number and distributed unevenly, thus making it difficult to produce accurate spatial distribution solely by interpolating the ground observations. Moreover, observations also suffer from a representativeness error. With the assistance of relevant auxiliary variables (e.g., satellite-derived Aerosol Optical Depth (AOD) data), the estimation accuracy of PM_2.5_ concentrations can be significantly improved. Various estimation models based on the relationship between PM_2.5_ and auxiliary variables have been proposed. These methods can be divided into two categories, i.e., physical models and statistical models. The physical models use atmospheric chemistry models to simulate the association between AOD and PM_2.5_, and then estimate PM_2.5_ using satellite-derived AOD and the derived association [10,11,12]. The statistical models apply statistical methods (e.g., multiple linear regression, generalized additive model, and random forest) to investigate the relationship between ground-measured PM_2.5_ and satellite-derived AOD and other auxiliary variables, and then builds an estimation model based on the derived relationship [13,14,15,16,17,18]. Most of the statistical models argued that PM_2.5_ concentration is affected by the selected auxiliary variables that are fixed throughout the estimation period. However, it has been reported that PM_2.5_ is sensitive to meteorological conditions, but this sensitivity changes over time [19,20,21]. For example, certain meteorological factors may not significantly affect PM_2.5_ concentration during a specific period [22]. Additionally, AOD is an optical remote sensing product, which is significantly influenced by weather conditions (e.g., cloud and rain), resulting in a large amount of data gaps. All of these factors inevitably have adverse effects on accurately estimating the spatial distribution of PM_2.5_ concentration.

Determining factors that influence PM_2.5_ concentration have recently generated increased research interest and involves various aspects, including economic development, natural conditions, and urbanization [23,24,25,26]. Although such research can provide useful references for formulating atmospheric environmental governance policies, it still has some shortages. It is common to represent PM_2.5_ concentration of a region based on one ground measurement [23,27,28]. Due to significant spatial variation in PM_2.5_ concentration, such simplification will likely corrode the reliability of results. Although some studies have estimated PM_2.5_ concentration in urban areas based on gridded PM_2.5_ data [29,30], these grid data mainly come from interpolation of ground PM_2.5_ observation data or from statistical models based on satellite-derived AOD. Nevertheless, it was not until February 2012 that China measured PM_2.5_ concentration as a proxy for environmental air quality and at the end of 2014 a national observation network to measure PM_2.5_ levels was established (~1500 observation stations). Therefore, the interpolation results and statistical model results before 2014 may have larger uncertainty due to the limitation of available observations. The relationship between PM_2.5_ concentration and influencing factors are complicated, which creates some uncertainty in model construction. Some studies have focused on the relationship from a time-series perspective without regarding the spatial dependence of PM**_2.5_** pollution [24,29,31]. Given the spatial dependence of atmospheric pollution, some studies applied spatial models to investigate the influencing factors on PM_2.5_ concentration [32,33]. However, the spatial model usually only considers panel data at a particular time, which may obtain different or even opposite results for panel data at different times because of the small sample data size. Although many studies have explored factors that influence PM_2.5_ concentration, it should be noted that at different stages of development, there can be substantial differences in economic growth, energy consumption, industrial structure, population, and environmental background. Ignoring these differences is very likely to cause biased or suspicious conclusions.

Given that the national observation network was not completed until the end of 2014, this study focused on the spatiotemporal evolution of PM_2.5_ concentration in 28 cities of the Beijing–Tianjin–Hebei region and surrounding areas during 2015–2017, and then identified its socioeconomic determinants. Firstly, an estimation model for high spatial-resolution PM_2.5_ estimation was created based on the reconstructed AOD missing gaps and the spatiotemporal heterogeneous relationship between PM_2.5_ and auxiliary variables, which disclosed the spatial distribution of PM_2.5_ concentration in the study area. Secondly, based on the analysis of the spatiotemporal evolution of PM_2.5_ concentration, the socioeconomic factors that influence local PM_2.5_ concentration were investigated by a spatial panel model. The research study’s conclusions provide scientific references for local atmospheric pollution control.

## 2. Study Area and Data

### 2.1. Study Area

**Figure 1:**
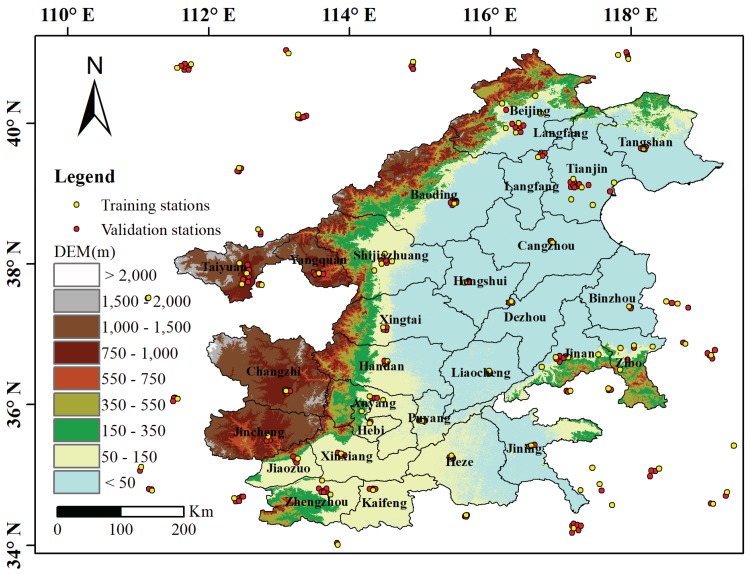
Study area and spatial distribution of ground PM 2.5 observation stations.

The study area includes 28 cities of the Beijing–Tianjin–Hebei region and surrounding areas, covering an area of ~275,000 km^2^. As shown in Figure 1, the terrain is high in the west and low in the east, with elevation ranging from sea level to >2000 m. The region experiences four distinct seasons, with hot and rainy summers due to the East Asian monsoon, and cold and dry winters due to subtropical high-pressure systems. The Beijing–Tianjin–Hebei region is the most economically developed area in northern China and is the area with the most PM_2.5_ pollution.

### 2.2. Data

Ground PM2.5 observations were from the public platform of the China National Environmental Monitoring Center (http://www.cnemc.cn). Before release, these data had been calibrated and quality controlled to meet the national environmental air quality standards of China (GB3095-2012). The present study used daily average PM_2.5_ concentration (DAPC) data from January 2015 to December 2017 at 256 observation stations in the study area and surrounding areas. Data of monthly average PM_2.5_ concentrations (MAPC) were generated by averaging the DAPC. Spatial distribution of observation stations is shown in Figure 1. Among 256 observation stations, 83 were used for constructing the PM_2.5_ estimation model, and the remaining were used to verify the accuracy of the estimation results. 

The latest C6 version of daily AOD (DAOD) was used to construct PM_2.5_ concentration estimation model. The C6 version DAOD had higher spatial resolutions (3 km) compared with the previous C5 version. Data of DAOD of Aqua (DAODA) and DAOD of Terra (DAODT) from January 2015 to December 2017 were collected from the website https://ladsweb.modaps.eosdis.nasa.gov/.

According to practical situations in the study area, air temperature (AT), wind speed (WS) at 10 m, surface pressure (SP), and boundary layer height (BLH) were chosen to assist the estimated spatial distribution of PM_2.5_ concentration (Table 1). These data came from the ERA-Interim reanalysis data (http://apps.ecmwf.int/datasets/) of the European Centre for Medium-Range Weather Forecast, with a spatial resolution of 0.125°.

Socioeconomic data were collected from the China Statistics Yearbook, China City Statistics Yearbook and statistical yearbooks of provinces and regions in the study area (http://data.cnki.net/Yearbook). Six factors were chosen: person density (PD), per capita gross regional product (PGRP), urbanization rate (UR), the proportion of secondary industry in GDP (PSIGDP), industrial smoke (dust) emissions (ISDE), and the number of industrial enterprises above designated size (NIEDS) (Table 1). All PGRP data were transformed uniformly to a constant price in 2015. Additionally, logarithmic transformations were performed on all socioeconomic data to eliminate their heteroscedasticity.

## 3. Methodology

### 3.1. PM_2.5_ Estimation

Due to the impact of clouds, rain, and other weather conditions, there are a lot of gaps (no data region) in DAOD data. Therefore, it was necessary to fill the missing data gaps. DAOD data were constructed based on the complementarity between DAODT and DAODA on spatial coverage and significant correlation. The data were filled as follows: 

DAODT and DAODA of a month were used to establish the relationship:

(1) $${\mathrm{DAODT}}_{m}=a_{T,m}+b_{T,m}{*\mathrm{DAODA}}_{,m}+\varepsilon_{T}$$

(2) $${\mathrm{DAODA}}_{m}=a_{A,m}+b_{A,m}{*\mathrm{DAODT}}_{m}+\varepsilon_{A}$$

where *m* refers to month. a_T,*m*_, b_T,*m*_, a_A,*m*_ and b_A,*m*_ are regression coefficients between DAODT and DAODA. ε_T_ and ε_A_ are error terms.

Next, the missing data gaps of DAODT and DAODA were reconstructed based on the acquired relationships in Equations (1) and (2), generating reconstructed DAODT (RDAODT) and reconstructed DAODA (RDAODA). For example, if DAODT has a value V_test_ at location L_test_, but DAODA does not, we can use V_test_ and Equation (2) to estimate the value of DAODA at location L_test_. In this way, DAOD was estimated as (RDAODT + RDAODA)/2 because Aqua and Terra measure AOD in the morning and afternoon, respectively. Finally, the monthly DAOD averages were calculated, which were used to generate the monthly average AOD data (MAOD).

Although AOD is an indicator of PM_2.5_ concentration, PM_2.5_ concentration is also significantly influenced by air temperature, precipitation, and other climatic factors [34,35]. This study used MAOD, AT, WS, BLH, and SP as the auxiliary variables to estimate the spatial distribution of PM_2.5_ concentration. We assumed that the relationship between PM_2.5_ concentration and auxiliary variables changes with time and space and the following model was constructed:

(3) $${\hat{\mathrm{MAPC}}}_{m}\left(u\right)=\alpha_{m}\left(u\right)+\beta_{m,0}\left(u\right){*\mathrm{MAOD}}_{m}\left(u\right)+\overset{n}{\underset{i=0}{\sum }}\beta_{m,i}\left(u\right){*\mathrm{AUX}}_{m,i}\left(u\right)$$

where ${\hat{\mathrm{MAPC}}}_{m}$ is the estimated average PM_2.5_ concentration during the month *m*. *u* refers to spatial position. α*_m_* is intercept, and *β_m_*_,0_ and *β_m_*_,*i*_ are coefficients of ${\mathrm{MAOD}}_{m}$ and other auxiliary variables AUX*_m_*_,*i*_. *n* is a variable with a value range of < =4. When *n* = 0, no climatic factor is chosen. When *n* = 4, the AT, WS, BLH, and SP were all used to construct the model.

With regards to the temporal heterogeneous relationship between PM_2.5_ and auxiliary variables, the auxiliary variables of the model were chosen based on the following criteria with consideration to temporal changes of the relationship between PM_2.5_ and auxiliary variables: (1) the chosen auxiliary variables were significantly correlated with PM_2.5_; (2) the chosen auxiliary variables improved the interpretation of the model to PM_2.5_ variation. In the views of the spatial heterogeneous relationship between PM_2.5_ and auxiliary variables, a local regression method, geographically weighted regression (GWR) [36], was applied to assess and describe the relationship. 

For each month, observations of the training stations were used to construct the PM_2.5_ estimation model, and observations from the validation stations were used to validate the accuracy of the estimated result (Figure 1). Some statistical indexes, including correlation coefficient, mean absolute error (MAE), and root-mean-square error (RMSE), were chosen to evaluate the effectiveness of the proposed model.

### 3.2. Effect of Economic and Social Factors on PM_2.5_ Concentration

Socioeconomic data from the statistical yearbook were based on city-scale annual statistics. Hence, the derived MAPC data should be processed accordingly, which generated the city-scale annual average PM_2.5_ concentration (AAPC). The logarithms of AAPC were calculated to ensure consistency with the pre-processing of Socioeconomic data.

The PM_2.5_ distribution presented strong trans-regional characteristics and inevitably affected nearby regions. Global Morans’ I analysis [37] was used to measure the spatial correlation of PM_2.5_ concentrations. In addition, local Morans’ I analysis [37] was applied to describe the spatial heterogeneity of PM_2.5_ concentrations in different geographical units.

The effects of socioeconomic factors on PM_2.5_ concentration in the urban scale were analyzed by a spatial panel model [38,39]:

(4) $$\begin{array}{c} lny_{it}=\rho \overset{N}{\underset{j=1}{\sum }}w_{ij}y_{jt}+\varphi +lnX_{it}\beta +\overset{N}{\underset{j=1}{\sum }}w_{ij}lnX_{jt}\gamma +\mu_{i}+\eta_{t}+\phi_{it}\\ \phi_{it}=\lambda \overset{N}{\underset{j=1}{\sum }}w_{ij}\phi_{it}+\varepsilon_{it},\varepsilon_{it}\sim N\left(0,\delta^{2}\right) \end{array}$$

where *i* refers to city and *t* is the year. *y_it_* is the explained variable, which is equal to AAPC of city *i* in year *t*. *ln*X*_it_* is the explanatory variable which refers to socioeconomic factors, and *β* is the corresponding coefficients. *µ_i_* is the spatial effect and *η_t_* is the time-period effect. *w_i,j_* refers to elements in the spatial weight matrix W. *ρ* is the spatial autoregression coefficient of dependent variables. *γ* denotes the spatial autocorrelation vector of explanatory variables. *λ* is the spatial autocorrelation coefficient of the error term.

When *γ* = *λ* = 0, the Equation (4) is simplified to a spatial panel lag model (SPLM):

(5) $$lny_{it}=\rho \overset{N}{\underset{j=1}{\sum }}w_{ij}+\varphi +lnX_{it}\beta +u_{i}+\eta_{t}+\varepsilon_{it},\varepsilon_{it}\sim N\left(0,\delta^{2}\right)$$

When *ρ*=*γ*=0, the Equation (4) is simplified to a spatial panel error model (SPEM):

(6) $$\begin{array}{c} lny_{it}=\varphi +lnX_{it}\beta +u_{i}+\eta_{t}+\phi_{it}\\ \phi_{it}=\lambda \overset{N}{\underset{j=1}{\sum }}w_{ij}\phi_{it}+\varepsilon_{it},\varepsilon_{it}\sim N\left(0,\delta^{2}\right) \end{array}$$

When *λ*=0, the Equation (4) is simplified to a spatial panel Dubin model (SPDM):

(7) $$lny_{it}=\rho \overset{N}{\underset{j=1}{\sum }}w_{ij}y_{jt}+\varphi +lnX_{it}\beta +\overset{N}{\underset{j=1}{\sum }}w_{ij}lnX_{jt}\gamma +u_{i}+\eta_{t}+\varepsilon_{it},\varepsilon_{it}\sim N\left(0,\delta^{2}\right)$$

## 4. Results and Discussion

### 4.1. Construction of the Estimation Model

Auxiliary variables were chosen monthly according to the selection criteria in Section 3.1 (Table 2). The estimation models of all months involved MAOD, which again confirms that AOD was a good indicator of PM_2.5_ concentration. The number of other chosen auxiliary variables changes with time, indicating that although PM_2.5_ concentration was greatly affected by the climatic conditions, there was a significant temporal change in sensitivity. This validates the justifiability of the proposed assumption. Many studies [6,40] have reported that precipitation affects PM_2.5_ concentration, and the effect is more significant in the time dimension or in the large spatial range. However, in this study, we constructed an estimation model for each month, which reduces the effect in the time dimension. Next, unlike other statistical methods, GWR is a local spatial regression method—only the data within the local range participates in the model construction, thereby weakening the effect in the large spatial range. As a result, precipitation was excluded in this study. This is consistent with other studies [41,42,43] that build PM_2.5_ estimation models based on GWR.

**Figure 2:**
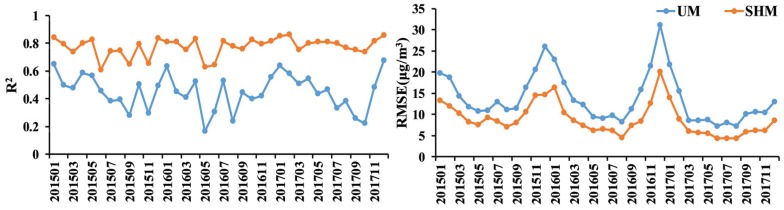
R^2^ and root-mean-square error (RMSE) values of the derived uniform relationship model (UM) and spatiotemporal heterogeneous model (SHM) for MAPC over the study area during 2015–2017.

The proposed spatiotemporal heterogeneous model (SHM) was compared with the uniform relationship model (UM) based on multiple linear regression. The construction accuracy of the estimation model throughout the study period is shown in Figure 2. UM showed a relatively lower goodness of fit and high temporal fluctuation, with minimum and maximum values of R^2^ being 0.17 and 0.67, respectively. Comparatively, SHM increased interpretation to changes in PM_2.5_ concentration. Its average of R^2^ (0.77) was significantly higher than that of UM (0.45), while its average of RMSE (8.87 μg/m^3^) was considerably smaller than that of UM (13.81 μg/m^3^). In addition, SHM demonstrated better stability. All these indicate that it is necessary to consider the spatial heterogeneity of the relationships between PM_2.5_ and auxiliary variables.

### 4.2. Accuracy Validation and Estimation Results

**Figure 3:**
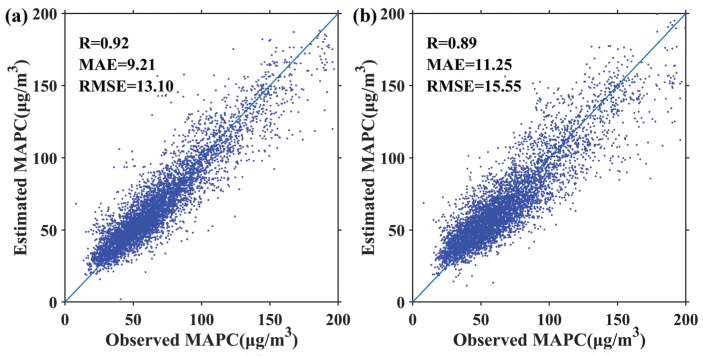
Scatterplots between estimated MAPC and ground observation by using SHM (**a**) and UM (**b**) over the study area during 2015–2017.

**Figure 4:**
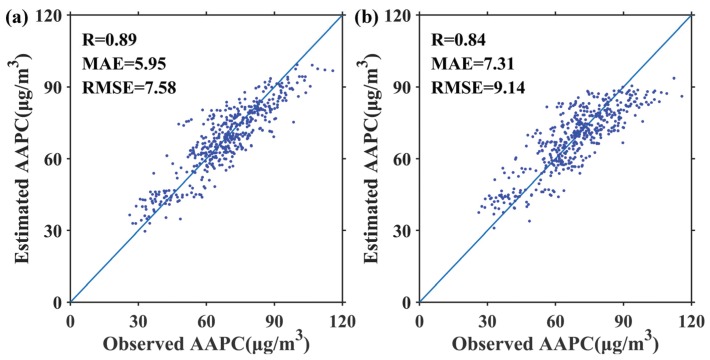
Scatterplots between estimated annual average PM_2.5_ concentration (AAPC) and ground observation by using SHM (**a**) and UM (**b**) over the study area during 2015–2017.

The validation of estimated MAPC in the study area during 2015–2017 is shown in Figure 3. R, MAE and RMSE of UM were 0.89, 11.25 μg/m^3^, and 15.55 μg/m^3^, respectively. In contrast, SHM significantly increased the estimation accuracy of MAPC, increasing the correlation coefficient by 3% and decreasing MAE and RMSE by as much as 18% and 16%, respectively. As expected, the proposed model achieved a higher estimation accuracy of AAPC than UM. The correlation coefficient of the proposed model increased by 5%, while the MAE and RMSE decreased by as much as 19% and 17%, respectively (Figure 4). Huang et al. [44] estimated 1 km MAPC in North China from 2013–2015, with RSME of 14.89 μg/m^3^. Ma et al. [45] produced China’s 10 km MAPC from 2014 to 2017 using a two-stage statistical model, with R^2^ ranging from 0.75 to 0.81. Wei et al. [46] estimated China’s 1 km MPAC in 2016 by using a space-time random forest approach, with R^2^ of 0.73 and RMSE of 14.88 μg/m^3^. Therefore, the overall accuracy of SHM is satisfying.

**Figure 5:**
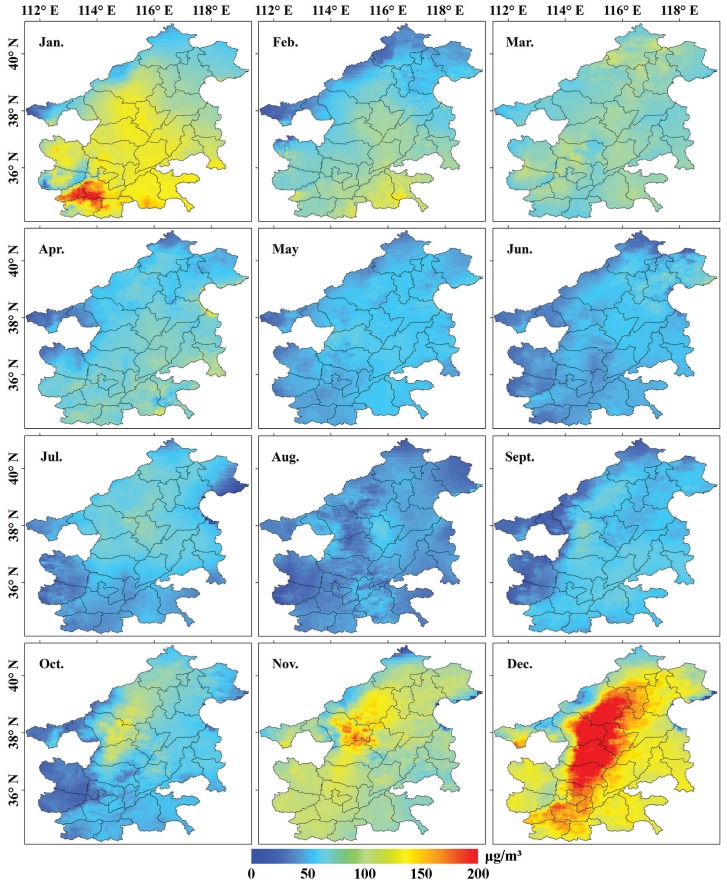
Spatial distribution of MAPC in each month of 2016.

**Figure 6:**
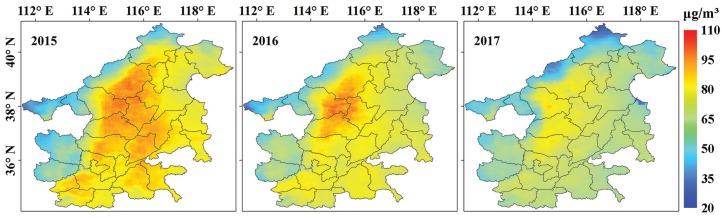
Spatial distribution of AAPC in 2015, 2016, and 2017.

Taking MAPC in 2016 as examples, their spatial distributions are shown in Figure 5. There is a significant spatiotemporal variation in PM_2.5_ concentration. In terms of spatial variation, high MAPC in the southeast region of the study area from January–April was observed, whereas PM_2.5_ concentration in the northwest region towards the central area was relatively higher from May–December. With regards to temporal variation, PM_2.5_ concentrations in January, February, November, and December were significantly higher than those in other months. The reason behind this may be related to indoor heating and climatic conditions. Some studies have reported that the burning of biomass and fossil energy for heating in winter generated huge PM_2.5_ emissions [47,48]. Dust storms, which frequently occur in North China in late winter and spring, is another major contribution that aggravates PM_2.5_ concentrations [49]. The lowest average MAPC (40.33 μg/m^3^) occurred in August, and the highest (138.49 μg/m^3^) was in December. The AAPC in the study area during 2015–2017 is shown in Figure 6. Generally, AAPC decreased from the central areas of Shijiazhuang, Baoding, Hengshui, and Xingtai to surrounding areas, accompanied with obvious concentration characteristics. The average APPC decreased from 77.3 μg/m^3^ in 2015 to 64.85 μg/m^3^ in 2017. 

### 4.3. Spatiotemporal Analysis of City-Scale PM_2.5_ Concentration

**Figure 7:**
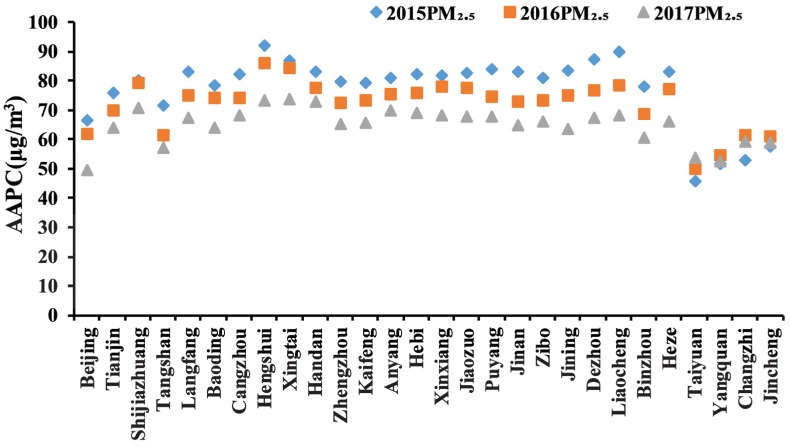
AAPC of 28 cities during 2015–2017.

Spatiotemporal variations of city-scale AAPC in the study area are shown in Figure 7. The AAPC in Taiyuan, Yangquan, Changzhi, and Jincheng changed slightly, but AAPC in the other cities decreased year by year, especially in Hengshui, Liaocheng, Dezhou, and Jinan. This might be related to the relatively high PM_2.5_ pollution present in these cities. Nevertheless, AAPC in all cities were still higher than the national environmental air quality standard of 35 μg/m^3^ (GB3095-2012), and much higher than the health standard recommended by the WHO of 10 μg/m^3^. This demonstrated that more work is required to control PM_2.5_ concentrations in the study area.

**Figure 8:**
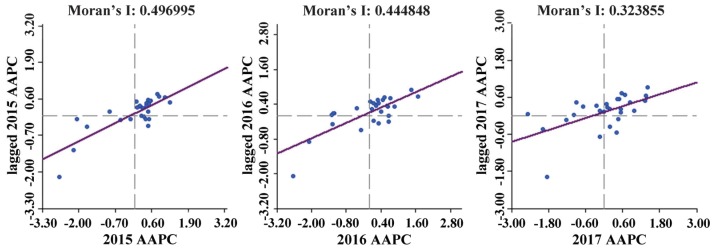
Global Moran’s I scatterplots of AAPC of 28 cities in 2015, 2016, and 2017.

Figure 8 shows the spatial correlation of city-scale AAPC in the study area. Global Moran’s I indexes were positive during 2015–2017 and all passed the significance test of 0.05, indicating that AAPC had significant spatial positive correlation and evident spatial concentration. Moreover, the Global Moran’s I decreased gradually, indicating that the concentration degree of AAPC decreased year by year.

**Figure 9:**
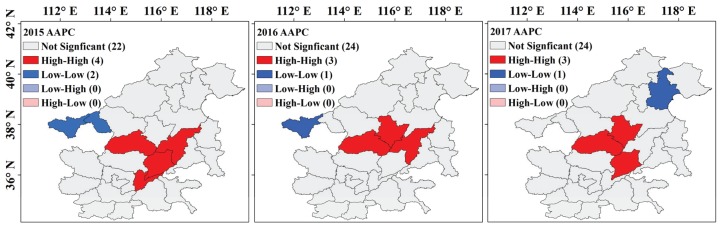
Local indicators of spatial association maps of city-scale AAPC for 2015, 2016, and 2017.

To further identify the local aggregation pattern of city-scale AAPC, the local Moran’s I analysis was performed (Figure 9). The AAPC was dominated by high-high (HH) and low-low (LL) aggregation types; however, high-low (HL) or low-high (LH) types were not found. This indicated that AAPC in the study area had a local spatial positive correlation. HH-type regions, also known as the high-value aggregation region of AAPC, stably locate in the study area center, whereas the distribution of LL-type regions is unstable. The LL-type regions were concentrated in the west of the study area during 2015–2016, but in the northeast region of the study area in 2017.

### 4.4. Effects of Socioeconomic Factors on PM_2.5_ Concentration

A series of tests were required to select the optimal analysis model. Firstly, to determine whether the spatial panel model should be applied, the Lagrange Multiplier (LM), and robust LM (RLM) tests were applied to assess the spatial correlation of the errors of the classic panel model. The LM error, LM lag, and RLM error under each condition (unfixed effects, spatial fixed effects, time-period fixed effects, and spatial and time-period fixed effects) all passed the 5% significance test (Table 3), indicating a significant spatial correlation of the errors in the classic panel model. Hence, the spatial panel model should be applied. The likelihood ratio (LR) tests of spatial fixed effects and time-period fixed effects both exceeded the 1% significance level, which proved the superiority of spatial and time-period fixed effects to the spatial fixed effects or time-period fixed effects. Subsequently, a spatial panel Durbin model with spatial and time-period fixed effects was constructed to test whether it could be simplified into SPLM or SPEM (Table 4). The Hausman test was significant at the 1% level, indicating that the model with random effects was rejected; the Wald and LR tests were significant at the 1% level. Therefore, the spatial panel Dubin model could not be simplified. 

The spatial panel Dubin model with spatial and time-period fixed effects was chosen to analyze how economic and social factors influence PM2.5 concentration. As shown in Table 5, UR has a significantly negative effect on PM_2.5_ concentration, and increasing the UR of a city by 1% could decrease PM_2.5_ concentration in the city by 0.78%. This is because, with the increasing demands for people to live in a heathy environment, China has implemented stricter environmental regulations than before. Considering the spatial interaction of UR, the PM_2.5_ concentration in a city could decrease by 2.23% if the UR in the surrounding cities is increased by 1%. Our findings are different from studies before 2010 [50], which suggested that UR has a positive effect on PM_2.5_ concentration. The reason behind this is that after China made carbon emission reduction commitments at the 2009 Copenhagen Climate Conference, the government, enterprises, and society have been vigorously promoting ecological sustainability, which effectively curbed the aggravation of air pollution, and reduced PM_2.5_ concentration.

The coefficients of *ln*PGRP and (*ln*PGRP)^2^ were significantly positive and negative, respectively, indicating the existence of an inverted U-shaped environmental Kuznets curve (EKC) of PM_2.5_ concentration in the study area. With the increase of the per capita income level, the study area experienced a process of pollution first and then treatment, resulting in the PM_2.5_ concentration first increasing and then decreasing, which is consistent with some existing research [51,52,53]. Although most cities in the study area except for Beijing, Tianjin, and Qinhuangdao are still in the stage of industrialization [54] and have a secondary industry-dominated structure, some cities shut down many small-sized, high-pollution, and high-energy-consumption enterprises in order to meet environmental quality requirements, and reached the peak of pollution ahead of schedule at the cost of low and medium economic growth. This is further demonstrated by the insignificant coefficient of PSIGDP.

Nevertheless, NIEDS has a significant positive effect on PM_2.5_ concentrations. PM_2.5_ concentrations in cities may increase by 0.15% and adjacent areas by 0.67% when NIEDS is increased by 1%. This is related to the fact that many NIEDS are resource- and energy-consuming enterprises. For example, the Hebei Iron and Steel Group is an ultra-large iron and steel group that ranks as the first in China and the second in the world in terms of crude steel output. Such enterprises provide important support for local employment and economic development. Given the path-dependence of their development, it is difficult to carry out energy saving and emission reduction measures immediately and thoroughly [55]. These enterprises will generate a large amount of dust pollution during production activities. Accordingly, the PM_2.5_ concentration of a city is increased 0.043% when the ISDE of adjacent cities is increased by 1%.

In addition to the above socioeconomic factors, the PM_2.5_ concentration of a city was also influenced by those in surrounding cities. The coefficient of the spatial lag term of PM_2.5_ concentration was 0.6337 and passed the 1% significance test, which was mainly due to the transmission and diffusion of PM_2.5_. Wang et al. [56] also reported that the contribution rate of foreign sources to PM_2.5_ concentrations in the Beijing–Tianjin–Hebei region was 23.4%.

To further identify the influence of different social factors, we calculated the direct, indirect, and total effects of socioeconomic factors on PM_2.5_ concentration (Table 6). Among seven factors, the total effect of *ln*PGRP, (*ln*PGRP)^2^, *ln*UR, *ln*ISDE, and *ln*NIEDS passed the significance test, indicating that these five factors influenced the spatiotemporal distribution of PM_2.5_ concentration in the study area. The order of the degree of influence for these five factors was: *ln*PGRP > *ln*UR > *ln*NIEDS > (*ln*PGRP)^2^ > *ln*ISDE. The other factors may not significantly influence PM_2.5_ concentration. To be specific, *ln*PGRP and *ln*UR are primary factors that influenced PM_2.5_ concentration, and the *ln*PGRP, *ln*UR, and *ln*NIEDS have spillover effects on the PM_2.5_ concentration in surrounding cities.

## 5. Conclusions

This study investigated the effects of socioeconomic factors on the spatiotemporal distribution of PM_2.5_ in Beijing–Tianjin–Hebei and surrounding areas during 2015–2017. First, an estimation model considering spatiotemporal heterogeneous relationships was developed to depict the spatiotemporal pattern of PM_2.5_ concentration in the study area. Then, on the basis of analyzing the spatiotemporal evolution of PM_2.5_ concentration, a spatial panel Dubin model was applied to determine how socioeconomic factors affect PM_2.5_ concentration. Major conclusions of this research include:

1. There is a significant spatiotemporal heterogeneous relationship between PM_2.5_ and the chosen auxiliary variables. The developed model can well estimate the spatial distribution of PM_2.5_ concentration in the study area, with MAE and RMSE of 9.21 μg/m^3^ and 13.1 μg/m^3^, respectively.
2. PM_2.5_ concentration in the study area showed significant spatial and temporal changes. Although PM_2.5_ concentration has decreased year by year, it was still higher than the national quality standard. Thus, further reduction in PM_2.5_ concentration remains a huge challenge.
3. PGRP, UR, and NIEDS were the key factors influencing the spatiotemporal distribution of PM_2.5_ concentration in the study area. Specially, there was an inverted U-shaped relationship between PGRP and PM_2.5_ concentrations. In addition, the increase of UR in a city will reduce PM_2.5_ concentration not only in its own city but in neighboring cities, while the increase of NIEDS of a city will exacerbate PM_2.5_ concentration in its own city and neighboring cities.

## Figures and Tables

**Table 1:** Definition of the variables used in the study.

Variable	Definition	Unit
AT	Air temperature at 2 m	K
WS	Wind speed at 10 m	m/s
BLH	Boundary layer height	m
SP	Surface pressure	Pa
PD	person density	person/km^2^
PGRP	Per capital gross regional product	yuan
UR	Urbanization rate	%
PSIGDP	The proportion of secondary industry in GDP	%
ISDE	Industrial smoke (dust) emissions	ton/year
NIEDS	The number of industrial enterprises above designated size	unit

**Table 2:** Variable selection of monthly average PM_2.5_ concentration (MAPC) estimation model for 2015–2017.

Month	Monthly Average AOD Data (MAOD)	AT	WS	BLH	SP
201501	√				
201502	√	√			
201503	√				√
201504	√				√
201505	√	√			
201506	√	√			
201507	√	√	√		√
201508	√		√		√
201509	√				√
201510	√	√			√
201511	√				√
201512	√	√	√		√
201601	√	√			√
201602	√			√	√
201603	√	√	√		√
201604	√	√		√	√
201605	√	√			√
201606	√	√			√
201607	√	√	√		√
201608	√			√	√
201609	√	√			√
201610	√		√		
201611	√		√	√	
201612	√		√		
201701	√		√	√	√
201702	√	√		√	
201703	√	√	√		
201704	√	√			√
201705	√	√		√	
201706	√	√			√
201707	√		√		
201708	√		√		
201709	√		√		
201710	√		√	√	
201711	√		√	√	
201712	√			√	√

**Table 3:** Diagnostic tests for non-spatial panel model.

Diagnostic Tests	No Fixed Effects (FE)	Spatial FE	Time FE	Two-Way FE
LM test spatial error	15.9629 ***	23.8827 ***	11.4793 ***	23.9171 ***
RLM test spatial error	8.4256 ***	27.7504 ***	6.4073 **	16.7844 ***
LM test spatial lag	7.6638 ***	5.0589 **	5.3875 **	13.1293 ***
RLM test spatial lag	0.1265	8.9266 ***	0.3154	5.9966 **
LR test		182.6997 ***	10.5856 **	

Note: ***, ** indicate significance at the 1%, 5% levels, respectively.

**Table 4:** Diagnostic tests for SPDM with two-way FE.

Diagnostic Tests	Statistics
Hausman test	148.1871 ***
Wald test spatial lag	27.9485 ***
LR spatial lag	25.0216 ***
Wald test spatial error	35.8282 ***
LR spatial error	29.7859 ***

Note: *** represent significance at the 1% levels, respectively.

**Table 5:** Estimation results of SPDM with two-way FE.

	Coefficient	*t* Value		Coefficient	*t* Value
*ln*PD	−0.0141	−0.5963	W*lnPD	−0.0243	−0.5542
*ln*PGRP	0.7351 *	0.8572	W*lnPGRP	2.7496 *	1.7305
(*ln*PGRP)^2^	−0.0332 *	−0.8595	W*(*ln*PGRP)^2^	−0.1359 *	−1.7796
*ln*UR	−0.7856 **	−2.1325	W*lnUR	−2.2324 ***	−3.0512
*ln*PSIGDP	−0.1035	−1.1761	W* *ln*PSIGDP	0.3455	1.8127
*ln*ISDE	0.0095	0.7338	W* *ln*ISDE	0.0432 *	1.9087
*ln*NIEDS	0.1491 *	1.7273	W* *ln*IEDS	0.6736 ***	2.6974
			W*dep.var.	0.6337 ***	7.6980

Note: ***, ** and * represent significance at the 1%, 5% and 10% levels, respectively.

**Table 6:** Decomposed spatial effects of SPDM with two-way FE.

	Direct Effects	*t* Value	Indirect Effects	*t* Value	Total Effects	*t* Value
*ln*PD	−0.0219	−0.6611	−0.0798	−0.6100	−0.1017	−0.6452
*ln*PGRP	1.6783 *	1.4878	8.2446 *	1.7204	9.9229 *	1.7623
(lnPGRP)^2^	−0.0790 *	−1.5492	−0.4014 *	−1.8354	−0.4804 *	−1.8706
*ln*UR	−1.5655 ***	−3.0675	−6.8348 ***	−2.9502	−8.4003 ***	−3.0936
*ln*PSIGDP	−0.0313	−0.2692	0.6839	1.2956	0.6527	1.0645
*ln*ISDE	0.0234	1.3370	0.1248 *	1.7119	0.1482 *	1.7148
*ln*NIEDS	0.3638 **	2.1617	1.8965 **	2.4697	2.2603 **	2.4722

Note: ***, ** and * represent significance at the 1%, 5%, and 10% levels, respectively.
